# Update of Emerinopathies’ clinical-genetic spectrum: the French network experience

**DOI:** 10.1186/1750-1172-10-S2-O16

**Published:** 2015-11-11

**Authors:** France Leturcq, Rabah Ben Yaou

**Affiliations:** 1 AP-HP, Groupe Hospitalier Cochin-Broca-Hôtel Dieu, Laboratoire de biochimie et génétique moléculaire (LBGM), Paris, France

## 

Emerinopathies include diseases caused by *EMD* gene mutations localized on chromosome X and encoding emerin, an integral protein of the nuclear envelope. The most frequent emerinopathy is the X-linked Emery-Dreifuss muscular dystrophy (X-EDMD) that was first reported in the 60ths by Emery and Dreifuss[1]. The disease is characterized by muscle weakness and wasting usually with a humeroperoneal distribution in the first stages, early joint contractures involving Achilles tendons, elbows, neck and the whole spine, and cardiac involvement featuring conduction defects, arrhythmias, subsequent cardiomyopathy (usually dilated) and frequently responsible of sudden death. Bione et al.[2] identified the first mutations of *EMD* gene encoding emerin to be responsible of X-EDMD. These mutations usually lead to absence or reduced level of emerin in different tissues of affected males including skeletal muscle, skin, oral mucosa cells and lymphocytes as it is demonstrated by immunocytochemical and histochemical methods[3,4]. Female carriers exhibit mosaic expression patterns with usually normal emerin amounts[3]. These methods may thus be used in the diagnostic strategy of X-EDMD prior to *EMD* gene analysis.

In a recent study (unpublished work from the French network and LBGM) aiming to assess the diagnostic utility of emerin study by western blot on lymphoblastoïd cell lines, we looked at *EMD* mutation rate observed in a cohort of 269 male and female patients with variable emerin amounts. In male patients, absence or severe reduction of emerin (<5%) lead to *EMD* mutation identification in all cases, while moderate emerin amount reduction revealed *EMD* mutation in 75% of the patients. Interestingly, in all cases where emerin amounts were considered as normal, no *EMD* mutations were found. In female cases, all cases with emerin moderate or severe reduction harbored EMD mutation. When emerin is normal, *EMD* mutation was found in 58% of female cases. These results suggest that a diagnostic rate of 100% may be reached if emerin study by western blot is performed prior to EMD gene screening in male patients. Moreover, emerin gene mutations have been rarely observed in rare cases of isolated cardiac disease[5,6] and limb girdle muscular dystrophies with cardiac disease and without joint contractures[7,8].

We reported of a new family with an unusual type of X-linked fatal isolated cardiac disease. The family included 9 affected male subjects with early death within the 4^th^ to 6^th^ decades and suffering from dilated cardiomyopathy (DCM) with arrhythmias requiring ICD implantation and or heart transplantation in some cases. Two surviving brothers were assessed. The first brother had DCM since 49 years old, ICD implantation at 51, additional Pacemaker at 52. His neurological assessment as well as CPK and EMG were normal at 53. His young brother had also DCM since 38 years old and his neurological assessment was normal at 45. Muscle biopsy performed in the oldest brother was considered as normal. Emerin protein analysis on muscle by Western blot using MANEM8 antibodies showed normal amounts while emerin immunostaining studies on muscle cryosections using NCL-Emerin antibodies showed complete absence of emerin. Subsequent *EMD* gene analysis revealed a missense mutation within exon 1.

By using the UMD-*EMD* locus specific database (http://www.umd.be/EMD/) gathering all published *EMD* gene mutations as well as those found in LBGM (more than 200 families), authors looked at *EMD* mutation spectrum. It was found that truncating mutations leading to absence or highly decreased emerin represent more than 65% of probands, while non-truncating mutations, including missense ones and leading to either absence or abnormal emerin, represents 21,5% of probands. The remaining probands (13.5%) carry intronic mutations with variable emerin amounts according to alternative splicing. There are no clear phenotype/genotype correlations due to high intra- and inter-familial variability. Truncating mutations may lead to classical forms of EDMD as well as severe forms with early ambulation loss and moderate forms with benign skeletal muscle involvement.
